# Association between the heart rate to temperature ratio and 28-day mortality in patients with heart failure in intensive care unit: a multicenter retrospective cohort study

**DOI:** 10.3389/fmed.2024.1497245

**Published:** 2024-12-13

**Authors:** Cheng Fu, Weiguo Lin, Xinglin Chen, WeiLi Hong, Shaorong Yan, Yuzhan Lin

**Affiliations:** ^1^Department of Clinical Laboratory, Ruian Traditional Chinese Medicine Hospital, Ruian, Zhejiang, China; ^2^Department of Urology, The Third Affiliated Hospital of Wenzhou Medical University, Ruian, Zhejiang, China; ^3^Department of Epidemiology and Biostatistics, Empower U, X&Y Solutions Inc., Boston, MA, United States; ^4^Department of Emergency Intensive Care Unit, The Third Affiliated Hospital of Wenzhou Medical University, Ruian, Zhejiang, China; ^5^Department of Clinical Laboratory, The Third Affiliated Hospital of Wenzhou Medical University, Ruian, Zhejiang, China

**Keywords:** heart failure, heart rate, temperature, mortality, intensive care unit

## Abstract

**Background:**

Heart failure (HF) is a life-threatening condition with a high mortality rate. The precise relationship between the heart rate and temperature (HR/T) ratio and mortality in patients with HF remains unclear. This study aimed to investigate the relationship between the HR/T ratio and 28-day intensive care unit (ICU) mortality rates in patients with HF.

**Methods:**

This retrospective cohort study analyzed the data of 3,790 patients with congestive heart failure in a large electronic database. Patients were divided into quartiles based on their HR/T ratio: Q1 (1.28–1.76), Q2 (2.44–2.72), Q3 (2.88–3.14), and Q4 (3.29–4.13). Multivariable logistic regression analysis was performed to examine the association between HR/T ratio and 28-day ICU mortality.

**Results:**

Patients with higher HR/T ratios had greater disease severity and higher mortality rates. In the fully adjusted regression model, a significant association was observed between HR/T ratio and 28-day ICU mortality risk, with mortality increasing as HR/T ratio rose (OR = 1.55, 95% CI: 1.17–2.04). An E-value analysis indicated that unmeasured confounders had a minimal impact on the results, confirming the robustness of the study.

**Conclusion:**

Among ICU-admitted patients with HF, we identified a significant association between HR/T ratio and 28-day ICU mortality. As the HR/T ratio increased, the 28-day ICU mortality showed an upward trend.

## 1 Introduction

Heart failure (HF) is a complex clinical syndrome defined by structural or functional abnormalities of the heart that result in elevated intracardiac pressure and insufficient cardiac output at rest or during physical activity (1). Globally, approximately 64.3 million individuals are affected by HF, and the prevalence of chronic HF is steadily increasing (2). In developed countries, the prevalence of HF in adults is approximately 1–2%, and is projected to increase to 3% by 2030 (3). Despite advancements in medical care, HF remains associated with high mortality rates, particularly in patients admitted to the intensive care unit (ICUs) (4). Therefore, an early evaluation of the clinical characteristics of patients with HF admitted to ICU is critical.

Excessive activation of the sympathetic nervous system is common in patients with HF and is one of the risk factors for mortality in these patients (5). As suggested earlier, the heart rate to temperature ratio (HR/T) can be used to assess sympathetic nervous system overactivation (6). The HR/T ratio, based on the heart rate and body temperature, is easy to measure in clinical practice and is an essential clinical indicator. Bistrovic et al. found that HR/T, an indicator of sympathetic overactivation, is associated with mortality in patients with coronavirus disease 2019 (COVID-19) (7). Additionally, the HR/T ratio correlates with ICU mortality in patients with sepsis (8). However, to the best of our knowledge, no previous studies have reported a relationship between the HR/T ratio and mortality in patients with HF admitted to ICU. Therefore, this study aimed to investigate the relationship between HR/T and 28-day ICU mortality in patients with HF admitted to ICU, using a publicly available large-scale eICU database.

## 2 Methods

### 2.1 Data source

This retrospective observational cohort study was based on the eICU Collaborative Research Database (eICU-CRD), an online international database in the United States (9). The eICU-CRD is a public database created through collaborative efforts between the MIT Laboratory for Computational Physiology and the eICU Institute (9). It contains high-resolution data of over 200,000 ICU admissions monitored across the United States as part of the eICU program from 2014 to 2015 (9). In accordance with the data use agreement of the PhysioNet review committee, the requisite examinations were completed and certification was obtained to enable access to the database (record ID: 40859994). In accordance with the Safe Harbor provision of the U.S. Health Insurance Portability and Accountability Act (HIPAA), the database utilized in this study was de-identified, and all protected health information was removed to ensure the safeguarding of institutional and patient privacy. This retrospective study did not involve direct patient intervention and was exempted from the safety protocol of the PhysioNet review committee. In light of the aforementioned reasons, the requirement for informed consent was not applicable. All methods used in this study were conducted in accordance with the tenets of the Declaration of Helsinki (10).

### 2.2 Study population

**Figure 1:**
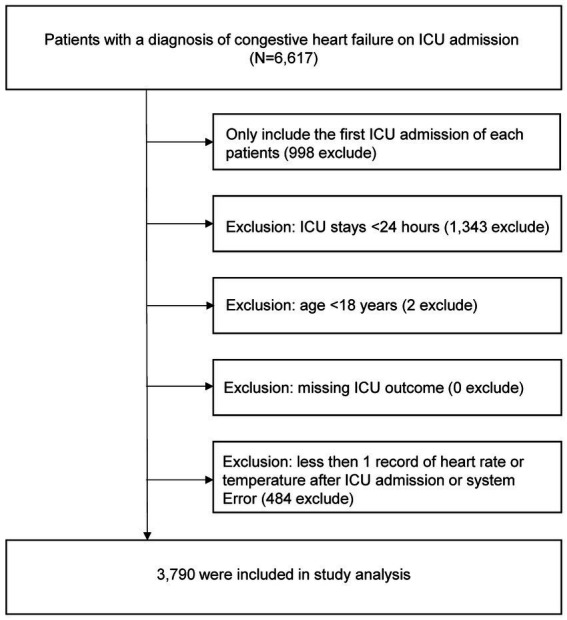
Flow chart of study population. ICU, intensive care unit.

The study population included all patients diagnosed with congestive heart failure (CHF) upon ICU admission. Specifically, patients were identified using the International Classification of Diseases, 9th Revision (ICD-9) code 428.0, as recorded in the database admission table (11). Study subjects were selected using the following exclusion criteria: (1) patients not on their first ICU admission, (2) patients with an ICU stay of less than 24 h, (3) patients under the age of 18 years, (4) patients lacking data monitoring during ICU treatment, and (5) patients with data having systematic errors or missing HR/T ratio after ICU admission. A flowchart of the inclusion and exclusion criteria is shown in Figure 1.

### 2.3 Main study variables

A Structured Query Language (SQL) was employed to extract data from the eICU-CRD. The extracted data included vital signs, such as heart rate, body temperature, respiratory rate (RR), and mean arterial pressure (MAP), which were recorded as the worst initial measurements within the first 24 h of ICU admission. The definition of the “worst” vital signs refers to the values that are furthest from the midpoint defined by Acute Physiology and Chronic Health Evaluation (APACHE; temperature = 38°C, heart rate = 75 beats per minute, RR = 19 breaths per minute, MAP = 90 mmHg) (9). The baseline characteristics of the patients, including sex, age, race, and body mass index (BMI), were collected from the patient and the patient result tables. Laboratory data, including alanine aminotransferase (ALT), aspartate aminotransferase (AST), Blood urea nitrogen levels (BUN), red blood cell count, hematocrit (Hct), and hemoglobin levels, were collected from the corresponding laboratory tables. The presence of comorbidities, including chronic obstructive pulmonary disease (COPD), acute myocardial infarction (AMI), liver failure, leukemia, and immunosuppression, was determined based on the APACHE IV scores. Treatment given at baseline included intubation, mechanical ventilation, dialysis, and *β*-blockers. The initial recorded value within the first 24 h of ICU admission was set as the baseline for analysis, using the measurement time as a reference point. The severity of the patients’ conditions upon admission was evaluated using several standardized scoring systems, including the Acute Physiology Score III, APACHE IV score, Glasgow Coma Scale (GCS) score, and Sequential Organ Failure Assessment (SOFA) score. The HR/T ratio was calculated by dividing the heart rate (beats per minute) by body temperature (°C). Patients were divided into quartiles based on their HR/T ratio: Q1 (1.28–1.76), Q2 (2.44–2.72), Q3 (2.88–3.14), and Q4 (3.29–4.13).

### 2.4 Outcomes

The primary outcome measure was 28-day mortality after ICU admission.

### 2.5 Statistical analyses

Statistical analyses were conducted using the software packages R (version 4.2.0, R Foundation for Statistical Computing, Vienna, Austria) and EmpowerStats (X&Y Solutions Inc., Boston, MA, United States).^1^ Descriptive statistics were used to summarize baseline patient characteristics. Continuous variables are presented as mean ± standard deviation (SD) or median with interquartile range (IQR). One-way analysis of variance (ANOVA) was used to ascertain discrepancies among the quartiles of the HR/T ratio in continuous variables. The Kruskal–Wallis test was used to evaluate differences in non-normally distributed continuous variables across groups. Categorical variables are expressed as absolute values and percentages, and differences were compared using the Chi-Square test or Fisher’s exact test, as appropriate.

To address potential confounding factors, three distinct adjustment models were constructed to assess the relationship between HR/T and 28-day mortality. The adjustment for covariates was based on the clinical significance of confounders (12) and a change of at least 10% in the matched odds ratio (OR) (13). The results are presented as OR with 95% confidence intervals (95% CI). The HR/T ratio was analyzed as a categorical variable based on quartiles (Q1-Q4), with the lowest quartile serving as the reference category. The *p* values for the trends across the HR/T ratio quartiles were calculated for all three models. Additionally, generalized additive models (GAM) were employed to illustrate the correlation between the HR/T ratio and mortality, with the findings presented through smoothed curve-fitting plots. We calculated the E-value to determine the potential influence of unmeasured confounders on the association between HR/T and 28-day ICU mortality (14). The E-value quantifies the extent to which unmeasured confounders potentially nullify the observed association between HR/T and 28-day ICU mortality. For all analyses, statistical significance was set at *p* < 0.05.

## 3 Results

### 3.1 Baseline characteristics of patients with HF in ICU

A total of 3,790 patients were categorized by HR/T ratio quartiles and comparison of demographic characteristics, vital signs, laboratory test results, disease severity, and comorbidities across different quartiles is presented in Table 1. Compared with patients in the lowest HR/T ratio quartile, those in Q4 were generally younger and exhibited a lower BMI, while sex and racial distributions were relatively balanced. Significant intergroup differences were observed in laboratory parameters, including AST, ALT, hemoglobin, and Hct, across the different HR/T ratio groups (*p* < 0.05).

**Table 1:** Baseline characteristics and 28-day mortality according to the quartiles of the HR/T ratio (*n* = 3,790).

	HR/T quartile				
	Q1	Q2	Q3	Q4	
	(1.28–1.76)	(2.44–2.72)	(2.88–3.14)	(3.29–4.13)	
Parameters	*N* = 948	*N* = 947	*N* = 947	*N* = 948	*p*-value
Demographics					
Age (years)	72.73 ± 12.07	69.41 ± 13.57	69.64 ± 13.61	69.17 ± 15.05	<0.001
Gender (N, %)					0.807
Male	465 (49.05)	470 (49.74)	458 (48.36)	479 (50.53)	
Female	483 (50.95)	475 (50.26)	489 (51.64)	469 (49.47)	
Ethnicity (N, %)					0.094
Caucasian	678 (72.20)	662 (70.58)	668 (71.29)	717 (76.11)	
African American	143 (15.23)	156 (16.63)	154 (16.44)	141 (14.97)	
Hispanic	63 (6.71)	48 (5.12)	45 (4.80)	30 (3.18)	
Asian	41 (4.37)	57 (6.08)	53 (5.66)	38 (4.03)	
Native American	6 (0.64)	4 (0.43)	5 (0.53)	4 (0.42)	
Other/Unknown	8 (0.85)	11 (1.17)	12 (1.28)	12 (1.27)	
BMI (kg/m^2^)	32.41 ± 10.57	32.52 ± 11.25	31.41 ± 9.93	30.71 ± 9.90	<0.001
Vital signs					
Heart rate (/min)	55.29 ± 8.71	93.92 ± 5.40	109.62 ± 5.14	135.12 ± 15.19	<0.001
Temperature	36.34 ± 0.55	36.47 ± 0.57	36.43 ± 0.61	36.38 ± 0.74	<0.001
HR/T (bpm/°C)	1.52 ± 0.24	2.58 ± 0.14	3.01 ± 0.13	3.71 ± 0.42	<0.001
Respiratory rate (bpm)	26.78 ± 14.61	28.94 ± 14.09	31.45 ± 14.13	33.65 ± 13.26	<0.001
MAP (mmHg)	63.00 (53.00–122.00)	64.00 (53.00–124.00)	66.00 (54.00–125.00)	65.00 (51.00–132.00)	0.003*
Laboratory data					
AST (U/L)	23.00 (16.00–37.00)	26.00 (19.00–46.50)	26.00 (18.00–46.75)	31.00 (21.00–54.00)	<0.001*
ALT (U/L)	20.00 (13.75–35.25)	25.00 (17.00–42.00)	24.00 (15.00–42.00)	24.00 (15.00–46.00)	<0.001*
Blood urea nitrogen (mg/dL)	33.00 (22.00–53.00)	31.00 (21.00–48.00)	29.00 (20.00–46.00)	30.00 (20.00–45.00)	<0.001*
Albumin (g/dL)	3.03 ± 0.53	3.01 ± 0.54	3.03 ± 0.57	3.03 ± 0.59	0.961
troponin – I (ng/mL)	0.12 (0.05–0.47)	0.18 (0.05–0.98)	0.15 (0.06–0.59)	0.17 (0.06–1.03)	0.009*
LDH (Units/L)	254.00 (228.50–318.50)	236.50 (194.00–308.50)	244.00 (188.00–314.00)	257.00 (217.00–302.00)	0.610*
Platelets (cells × 10^9^/L)	193.00 (150.00–245.00)	196.00 (151.00–249.00)	198.00 (151.00–254.00)	198.00 (152.00–255.00)	0.122*
RBC (M/mcl)	3.64 ± 0.73	3.71 ± 0.79	3.73 ± 0.79	3.82 ± 0.77	<0.001
Hemoglobin (g/dL)	10.52 ± 2.04	10.72 ± 2.19	10.86 ± 2.27	11.05 ± 2.22	<0.001
Hct (%)	32.69 ± 6.30	33.34 ± 6.75	33.61 ± 6.87	34.16 ± 6.63	<0.001
BNP (pg/mL)	970.00 (503.00–2411.95)	1165.70 (583.00–2926.60)	1017.00 (464.75–2765.60)	1215.00 (629.00–3104.00)	0.316*
myoglobin(ng/mL)	101.00 (82.97–118.00)	254.50 (84.03–397.70)	84.50 (75.50–94.20)	130.30 (123.75–400.40)	0.125*
Hospital admit source (N, %)					0.383
Emergency Department	467 (64.33)	421 (62.00)	391 (58.27)	389 (57.29)	
Operating Room	109 (15.01)	128 (18.85)	130 (19.37)	146 (21.50)	
Floor	59 (8.13)	54 (7.95)	72 (10.73)	64 (9.43)	
Direct Admit	4 (0.55)	7 (1.03)	4 (0.60)	3 (0.44)	
Other Hospital	2 (0.28)	1 (0.15)	0 (0.00)	4 (0.59)	
Other ICU	18 (2.48)	12 (1.77)	18 (2.68)	19 (2.80)	
ICU to SDU	33 (4.55)	29 (4.27)	33 (4.92)	26 (3.83)	
Chest Pain Center	30 (4.13)	24 (3.53)	22 (3.28)	22 (3.24)	
Unit admit source (N, %)					0.337
Emergency Department	619 (65.36)	590 (62.37)	561 (59.24)	555 (58.61)	
Operating Room	159 (16.79)	194 (20.51)	192 (20.27)	213 (22.49)	
ICU to SDU	66 (6.97)	72 (7.61)	93 (9.82)	78 (8.24)	
Floor	1 (0.11)	2 (0.21)	1 (0.11)	1 (0.11)	
Other Hospital	2 (0.21)	1 (0.11)	0 (0.00)	2 (0.21)	
Step-Down Unit (SDU)	41 (4.33)	32 (3.38)	37 (3.91)	31 (3.27)	
Chest Pain Center	25 (2.64)	21 (2.22)	32 (3.38)	32 (3.38)	
Recovery Room	30 (3.17)	31 (3.28)	30 (3.17)	28 (2.96)	
Treatment					
Intubated (N, %)					0.215
No	833 (87.87%)	843 (89.02%)	831 (87.75%)	814 (85.86%)	
Yes	115 (12.13%)	104 (10.98%)	116 (12.25%)	134 (14.14%)	
Mechanical ventilation use (N, %)					0.283
No	567 (59.81%)	569 (60.08%)	577 (60.93%)	604 (63.71%)	
Yes	381 (40.19%)	378 (39.92%)	370 (39.07%)	344 (36.29%)	
Dialysis (N, %)					0.746
No	883 (93.14%)	870 (91.87%)	878 (92.71%)	875 (92.30%)	
Yes	65 (6.86%)	77 (8.13%)	69 (7.29%)	73 (7.70%)	
*β*-blocker use (N, %)					<0.001
No	643(67.83)	688(72.65)	669(70.64)	611 (64.45)	
Yes	305(32.17)	259(27.35)	278(29.36)	337(35.55)	
Severity of illness					
Acute Physiology Score III (N, %)					<0.001
Low (0.0–34.0)	326 (37.60)	369 (42.12)	313 (36.02)	142 (16.53)	
Middle (35.0–47.0)	270 (31.14)	283 (32.31)	294 (33.83)	260 (30.27)	
High (48.0–163.0)	271 (31.26)	224 (25.57)	262 (30.15)	457 (53.20)	
Apache IV score (N, %)					<0.001
Low (12.0–48.0)	295 (34.03)	363 (41.44)	304 (34.98)	164 (19.09)	
Middle (49.0–64.0)	302 (34.83)	305 (34.82)	306 (35.21)	269 (31.32)	
High (65.0–176.0)	270 (31.14)	208 (23.74)	259 (29.80)	426 (49.59)	
GCS score (N, %)					0.023
Low (3.0–13.0)	226 (24.02)	192 (20.51)	187 (20.00)	223 (23.88)	
Middle (14.0–14.0)	133 (14.13)	131 (14.00)	103 (11.02)	114 (12.21)	
High (15.0–15.0)	582 (61.85)	613 (65.49)	645 (68.98)	597 (63.92)	
SOFA score (N, %)					<0.001
Low (0.0–1.0)	260 (27.43)	299 (31.57)	360 (38.01)	330 (34.81)	
Middle (2.0–3.0)	332 (35.02)	329 (34.74)	281 (29.67)	266 (28.06)	
High (4.0–16.0)	356 (37.55)	319 (33.69)	306 (32.31)	352 (37.13)	
Comorbidities (N, %)					
Hepatic failure					0.113
No	940 (99.16)	942 (99.47)	933 (98.52)	942 (99.37)	
Yes	8 (0.84)	5 (0.53)	14 (1.48)	6 (0.63)	
Metastatic cancer					0.007
No	945 (99.68)	936 (98.84)	937 (98.94)	929 (98.00)	
Yes	3 (0.32)	11 (1.16)	10 (1.06)	19 (2.00)	
Leukemia					0.045
No	941 (99.26)	946 (99.89)	938 (99.05)	937 (98.84)	
Yes	7 (0.74)	1 (0.11)	9 (0.95)	11 (1.16)	
Immunosuppression					<0.001
No	941 (99.26)	930 (98.20)	930 (98.20)	913 (96.31)	
Yes	7 (0.74)	17 (1.80)	17 (1.80)	35 (3.69)	
Cirrhosis					0.379
No	935 (98.63)	936 (98.84)	939 (99.16)	942 (99.37)	
Yes	13 (1.37)	11 (1.16)	8 (0.84)	6 (0.63)	
Diabetes					<0.001
No	575 (60.65)	587 (61.99)	633 (66.84)	679 (71.62)	
Yes	373 (39.35)	360 (38.01)	314 (33.16)	269 (28.38)	
COPD					0.347
No	816 (86.08)	808 (85.32)	788 (83.21)	807 (85.13)	
Yes	132 (13.92)	139 (14.68)	159 (16.79)	141 (14.87)	
CHF					0.545
No	153 (16.14)	154 (16.26)	146 (15.42)	169 (17.83)	
Yes	795 (83.86)	793 (83.74)	801 (84.58)	779 (82.17)	
AMI					0.023
No	915 (96.52)	888 (93.77)	897 (94.72)	889 (93.78)	
Yes	33 (3.48)	59 (6.23)	50 (5.28)	59 (6.22)	
Pneumonia					0.165
No	857 (90.40)	852 (89.97)	842 (88.91)	829 (87.45)	
Yes	91 (9.60)	95 (10.03)	105 (11.09)	119 (12.55)	
Rhythm					<0.001
No	945 (99.68)	943 (99.58)	943 (99.58)	943 (99.47)	
Yes	3 (0.32)	4 (0.42)	4 (0.42)	5 (0.53)	
ICU 28 day mortality (N, %)					<0.001
No	913 (96.31)	911 (96.20)	899 (94.93)	877 (92.51)	
Yes	35 (3.69)	36 (3.80)	48 (5.07)	71 (7.49)	

Data were expressed as the mean ± SD or median (interquartile range) for continuous variables and number (%) for categorical variables. P*: Kruskal Wallis test was used to compare non-normally distributed continuous variables between groups. BMI, body mass index; HR/T, heart rate/temperature; MAP, mean arterial pressure; AST, aspartate transaminase; ALT, alanine transaminase; LDH, lactate dehydrogenase; RBC, red blood cell; Hct, hematocrit; BNP, B-type natriuretic peptide; ICU, intensive care unit; SDU, Step-Down Unit; GCS, Glasgow Coma Scale; SOFA, Sequential Organ Failure Assessment; COPD, chronic obstructive pulmonary disease; CHF, congestive heart failure; AMI, acute myocardial infarction.

Overall, the 28-day ICU mortality rate was 5.01% (190/3790). Notably, the 28-day mortality rate increased significantly with a higher HR/T ratio, from 3.69% in Q1 to 7.49% in Q4 (*p* < 0.001).

### 3.2 Association between the HR/T ratio and 28-day ICU mortality

A univariate logistic model was used to demonstrate the unadjusted associations between the baseline variables and 28-day mortality in patients in ICU (Table 2). There was a statistically significant positive correlation between HR/T ratio and mortality risk in patients with HF (*p* < 0.001). With regard to the source of admission, patients in the operating room exhibited a higher mortality risk than those in the emergency department (*p* = 0.002). Regarding the assessment of disease severity, the Acute Physiology Score III, APACHE IV, and SOFA scores demonstrated an elevated risk of mortality.

**Table 2:** Effects of factors on the HR/T ratio by univariate analysis.

Exposure	Statistics	OR (95%CI)	*p*-value
Demographics			
Age (years)	70.24 ± 13.69	1.02 (1.01, 1.03)	0.001
Gender (N, %)			
Male	1872 (49.42)	Ref.	
Female	1916 (50.58)	1.04 (0.78, 1.40)	0.778
Ethnicity (N, %)			
Caucasian	2,725 (72.55)	Ref.	
African American	594 (15.81)	0.74 (0.48, 1.14)	0.170
Hispanic	186 (4.95)	0.28 (0.09, 0.87)	0.028
Asian	189 (5.03)	0.55 (0.24, 1.26)	0.159
Native American	19 (0.51)	0.93 (0.12, 7.04)	0.947
Other/Unknown	43 (1.14)	0.40 (0.05, 2.93)	0.367
BMI (kg/m^2^)	31.76 ± 10.45	1.01 (0.99, 1.02)	0.443
Vital signs			
Heart rate (/min)	98.48 ± 30.49	1.01 (1.01, 1.02)	<0.001
Temperature (°C)	36.41 ± 0.62	0.92 (0.73, 1.16)	0.467
HR/T (bpm/°C)	2.71 ± 0.84	1.45 (1.21, 1.73)	<0.001
Respiratory rate (bpm)	30.20 ± 14.26	1.02 (1.01, 1.03)	0.002
MAP (mmHg)	87.10 ± 42.09	0.99 (0.99, 1.00)	0.001
Laboratory data			
AST (U/L)	119.44 ± 659.76	1.00 (1.00, 1.00)	0.228
ALT (U/L)	93.64 ± 462.21	1.00 (1.00, 1.00)	0.213
Blood urea nitrogen (mg/dL)	36.95 ± 22.47	1.01 (1.01, 1.02)	<0.001
Albumin(g/dL)	3.03 ± 0.55	0.66 (0.47, 0.94)	0.012
troponin - I(ng/mL)	1.78 ± 6.91	1.02 (0.99, 1.04)	0.150
LDH(Units/L)	313.45 ± 245.90	1.00 (1.00, 1.00)	0.929
Platelets (cells × 10^9^/L)	208.63 ± 91.70	1.00 (1.00, 1.00)	0.202
RBC (M/mcl)	3.72 ± 0.77	0.97 (0.79, 1.19)	0.746
Hemoglobin (g/dL)	10.79 ± 2.19	0.99 (0.92, 1.06)	0.745
Hct (%)	33.45 ± 6.66	1.00 (0.98, 1.02)	0.973
BNP (pg/mL)	3455.02 ± 6948.42	1.00 (1.00, 1.00)	0.412
myoglobin(ng/mL)	382.48 ± 1208.26	1.00 (0.99, 1.01)	0.681
Hospital admit source (N, %)			
Emergency Department	1,668 (60.54)	Ref.	
Operating Room	513 (18.62)	1.91 (1.27, 2.88)	0.002
Floor	249 (9.04)	1.64 (0.94, 2.88)	0.084
Direct Admit	18 (0.65)	1.41 (0.18, 10.72)	0.742
Other Hospital	7 (0.25)	3.98 (0.47, 33.55)	0.204
Other ICU	67 (2.43)	1.93 (0.75, 4.95)	0.173
ICU to SDU	121 (4.39)	1.25 (0.53, 2.94)	0.614
Chest Pain Center	98 (3.56)	1.84 (0.82, 4.12)	0.139
Unit admit source (N, %)			
Emergency Department	2,325 (61.39)	Ref.	
Operating Room	758 (20.02)	1.81 (1.27, 2.57)	<0.001
ICU to SDU	309 (8.16)	1.79 (1.10, 2.92)	0.012
Floor	5 (0.13)	6.14 (0.68, 55.46)	0.106
Other Hospital	5 (0.13)	6.14 (0.68, 55.46)	0.106
Step-Down Unit (SDU)	141 (3.72)	1.28 (0.58, 2.82)	0.536
Chest Pain Center	110 (2.90)	1.67 (0.75, 3.69)	0.206
Recovery Room	119 (3.14)	2.25 (1.14, 4.45)	0.019
Treatment			
Intubated (N, %)			
No	3,321 (87.63)	Ref.	
Yes	469 (12.37)	2.63 (1.87, 3.70)	<0.001
Mechanical ventilation use (N, %)			
No	2,317 (61.13)	Ref.	
Yes	1,473 (38.87)	1.44 (1.08, 1.93)	0.014
Dialysis (N, %)			
No	3,506 (92.51)	Ref.	
Yes	284 (7.49)	0.39 (0.17, 0.89)	0.025
Severity of illness (N, %)			
Acute Physiology Score III			
Low (0.0–34.0)	1,150 (33.13)	Ref.	
Middle (35.0–47.0)	1,107 (31.89)	1.60 (0.98, 2.62)	0.062
High (48.0–163.0)	1,214 (34.98)	4.31 (2.81, 6.61)	<0.001
Apache IV score			
Low (12.0–48.0)	1,126 (32.44)	Ref.	
Middle (49.0–64.0)	1,182 (34.05)	2.65 (1.57, 4.45)	<0.001
High (65.0–176.0)	1,163 (33.51)	5.66 (3.49, 9.19)	<0.001
GCS score			
Low (3.0–13.0)	828 (22.10)	Ref.	
Middle (14.0–14.0)	481 (12.84)	0.54 (0.34, 0.87)	0.010
High (15.0–15.0)	2,437 (65.06)	0.34 (0.25, 0.48)	<0.001
SOFA score			
Low (0.0–1.0)	1,249 (32.96)	Ref.	
Middle (2.0–3.0)	1,208 (31.87)	1.61 (1.01, 2.57)	0.046
High (4.0–16.0)	1,333 (35.17)	3.80 (2.52, 5.73)	<0.001
Comorbidities (N, %)			
Hepatic failure			
No	3,757 (99.13)	Ref.	
Yes	33 (0.87)	0.59 (0.08, 4.33)	0.604
Metastatic cancer			
No	3,747 (98.87)	Ref.	
Yes	43 (1.13)	2.53 (0.99, 6.51)	0.054
Leukemia			
No	3,762 (99.26)	Ref.	
Yes	28 (0.74)	2.29 (0.69, 7.67)	0.177
Immunosuppression			
No	3,714 (97.99)	Ref.	
Yes	76 (2.01)	1.96 (0.89, 4.32)	0.096
Cirrhosis			
No	3,752 (99.00)	Ref.	
Yes	38 (1.00)	1.05 (0.25, 4.41)	0.943
Diabetes			
No	2,474 (65.28)	Ref.	
Yes	1,316 (34.72)	0.70 (0.50, 0.96)	0.030
COPD			
No	3,219 (84.93)	Ref.	
Yes	571 (15.07)	0.50 (0.30, 0.85)	0.010
AMI			
No	3,589 (94.70)	Ref.	
Yes	201 (5.30)	0.57 (0.25, 1.30)	0.181
Pneumonia			
No	3,380 (89.18)	Ref.	
Yes	410 (10.82)	2.09 (1.44, 3.04)	<0.001
Rhythm			
No	3,042 (80.26)	Ref.	
Yes	748 (19.74)	1.40 (1.00, 1.96)	0.051

Data are expressed as the mean ± SD, or number (%). OR, odds ratio; BMI, body mass index; HR/T, heart rate/temperature; MAP, mean arterial pressure; AST, aspartate transaminase; ALT, alanine transaminase; LDH, lactate dehydrogenase; RBC, red blood cell; Hct, hematocrit; BNP, B-type natriuretic peptide; ICU, intensive care unit; SDU, Step-Down Unit; GCS, Glasgow Coma Scale; SOFA, Sequential Organ Failure Assessment; COPD, chronic obstructive pulmonary disease; AMI, acute myocardial infarction; Ref., Reference.

A variety of covariate adjustment strategies were employed, and the results of the multivariate regression model showed an association between the HR/T ratio and 28-day ICU mortality (Table 3). When the HR/T ratio was treated as a continuous variable, the unadjusted model demonstrated a positive correlation between an increasing HR/T ratio and 28-day mortality (OR = 1.45, 95% CI: 1.21–1.73). After adjustment for gender, age, ethnicity, and mechanical ventilation use in Model 2, the results were similar to those of the unadjusted model (OR = 1.49, 95% CI: 1.25–1.79, *p* < 0.001). Building on adjusted Model 2, after accounting for additional confounding variables, including diabetes, CHF, COPD, MAP, AMI, pneumonia, rhythm, *β*-blocker use, SOFA score, BUN, Albumin, Hct, ALT, there was an enhanced association between the HR/T ratio and 28-day mortality in patients with HF, which remained statistically significant (OR = 1.55, 95% CI: 1.17–2.04, *p* = 0.002). Upon analysis of the HR/T ratio by quartile in adjusted Model 3, the highest quartile (Q4, 3.29–4.13) exhibited a markedly elevated mortality risk compared with the lowest quartile (Q1, 1.28–1.76; OR = 2.66, 95% CI: 1.34–5.30).

**Table 3:** Associations between the HR/T ratio and 28-day mortality.

	Model 1		Model 2		Model 3	
	OR (95% CI)	*p*-value	OR (95% CI)	*p*-value	OR (95% CI)	*p*-value
HR/T (bpm/°C)	1.45 (1.21, 1.73)	<0.001	1.49 (1.25, 1.79)	<0.001	1.55 (1.17, 2.04)	0.002
HR/T quartile						
Q1(1.28–1.76)	Ref.		Ref.		Ref.	
Q2(2.44–2.72)	1.03 (0.64, 1.66)	0.900	1.14 (0.70, 1.84)	0.596	1.71 (0.81, 3.59)	0.158
Q3(2.88–3.14)	1.39 (0.89, 2.17)	0.145	1.52 (0.97, 2.39)	0.068	2.01 (0.98, 4.14)	0.058
Q4(3.29–4.13)	2.11 (1.39, 3.20)	<0.001	2.30 (1.51, 3.51)	<0.001	2.66 (1.34, 5.30)	0.005
P for trend		<0.001		<0.001		0.005

Model 1: Non-adjusted model. Model 2 adjust for: Gender, Age, Ethnicity, Mechanical ventilation use. Model 3 adjust for: Gender, Age, Ethnicity, Diabetes, CHF, COPD, MAP, AMI, Pneumonia, Rhythm, Mechanical ventilation use, *β*-blocker use, SOFA score, BUN, Albumin, Hct, ALT.

**Figure 2:**
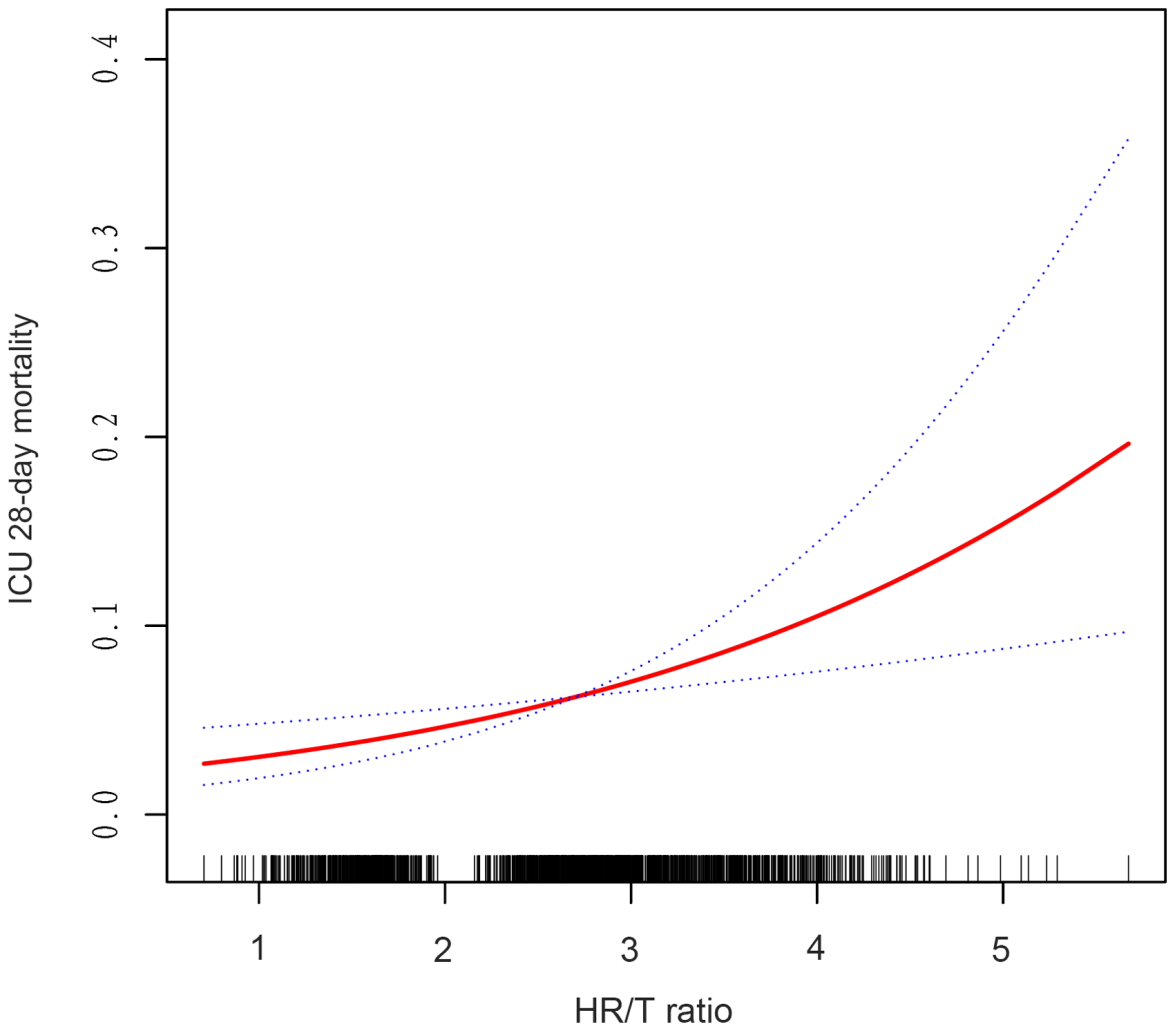
Associations between the HR/T ratio and 28-day mortality in all patients with heart failure. The solid rad line represents the smooth curve fit. The blue dashed line represents the 95% confidence interval. Adjusted for gender, age, ethnicity, diabetes, CHF, COPD, MAP, AMI, pneumonia, rhythm, mechanical ventilation use, *β*-blocker use, SOFA score, BUN, albumin, hct, ALT. HR/T, heart rate/temperature.

Using GAMs and curve fitting, an association between the HR/T ratio and 28-day ICU mortality in patients with HF remained apparent (Figure 2). After adjusting for covariates such as gender, age, ethnicity, diabetes, mechanical ventilation use, CHF, COPD, MAP, AMI, pneumonia, rhythm, *β*-blocker use, SOFA score, BUN, albumin, Hct, and ALT, a trend of increasing 28-day ICU mortality with rising HR/T ratios was observed. Additionally, the calculated E-value of 2.42 suggests stability and indicates that unmeasured confounders are unlikely to substantially affect the results.

## 4 Discussion

This study analyzed clinical data from 3,790 HF patients in the eICU database and identified a significant association between HR/T ratio and 28-day ICU mortality risk. Specifically, higher HR/T ratios correlated with an increased risk of 28-day ICU mortality. To the best of our knowledge, this study is the first to report the relationship between HR/T ratio and 28-day mortality in critically ill patients with HF. These findings provide a theoretical basis for the clinical importance of HR/T ratio in cardiovascular diseases and underscore its potential value in assessing the mortality risk for critically ill patients with HF admitted to the ICU.

Among the 3,790 patients with HF included in this study, 190 died, resulting in an overall mortality rate of 5%. The mortality rate in Q1 was relatively low (3.69%), suggesting that the proportion of patients with high disease severity scores in the Q1 group was lower compared to the Q4 group. Specifically, 31.14% of patients in the Q1 group had higher APACHE scores (270/867) than 49.59% in the Q4 group (426/859). Similarly, 31.26% of the patients in the Q1 group had high Acute Physiology Score III (271/867) compared to 53.20% in the Q4 group (457/859).

Research on the correlation between HR/T ratio and mortality is scarce. Prior research has demonstrated that the HR/T ratio is predominantly linked to unfavorable outcomes pertaining to inflammatory processes. Leibovici et al. first identified a significant association between the HR/T ratio and poor clinical outcomes in 3,382 patients with systemic inflammatory response syndrome, suggesting that a high HR/T ratio may serve as an independent risk factor for mortality (6). Similarly, Dmitri Guz et al. investigated the correlation between the HR/T ratio and 30-day mortality in 1,186 patients undergoing *β*-blocker therapy (15). Using a multivariable logistic regression model, their study found that patients with a higher HR/T ratio exhibited a 75.7% increased risk of all-cause mortality compared to those with a lower HR/T ratio (OR = 1.757, 95% CI: 1.069–2.886, *p* = 0.03). Bistrovic et al. expanded the clinical implications of the HR/T ratio by demonstrating that a low HR/T ratio in critically ill patients with COVID-19 appears to predict mortality, major bleeding, and the need for mechanical ventilation (7). Lin et al. identified an HR/T ratio threshold of 2.22 through a two-piece-wise linear regression model and recursive algorithm, that closely associated with higher ICU mortality in critically ill patients (8). Unlike previous studies, our research identified a correlation between the HR/T ratio and 28-day ICU mortality in patients with HF, providing a theoretical basis for further research in this area.

Heart rate and body temperature are closely associated with the pathophysiological mechanisms of HF, which involve intricate physiological systems and molecular pathways. HF is generally considered a consequence of compensatory adaptations in cellular, neurohumoral, and structural elements following cardiac injury, which elicit activation of the sympatho-adrenomedullary system (16). This initiates a cascade of compensatory physiological responses, culminating in volume overload, elevated HR, dyspnea, and further deterioration of cellular functions, thereby establishing a vicious cycle (17). The resting HR is determined by the activity of the sinoatrial node, the primary pacemaker of the heart, which is largely influenced by the interplay between sympathetic and vagal nerves (18). Accordingly, an elevated resting HR is indicative of an imbalance between sympathetic overactivity and reduced vagal tone (18). Furthermore, immune responses involving macrophages and other immune-active cells in the inflammatory state play a pivotal role in the pathophysiology of HF (19). In patients with HF, the activation of the sympathetic nervous system results in the desensitization of β2-adrenergic receptors, which in turn alters immune function (20). Moreover, inflammatory cytokines such as interleukin-6 have been linked to the pathophysiology of HF (21, 22). The cascade of these cytokines can stimulate immune cells, including macrophages, T cells, and monocytes, to release IL-17 and TNF-*α* (23). These cytokines function as signals that facilitate the differentiation of immune cells into proinflammatory and profibrotic subsets. These mechanisms result in an elevated HR during the compensatory phase of HF and a reduced HR during the decompensatory phase, which subsequently alters the HR/T ratio. This may elucidate the correlation between the HR/T ratio and 28-day mortality in patients with HF.

There are four primary limitations of the study. (1) The definition of the HR/T ratio has some issues; the eICU-CRD only provides the worst HR and temperature within the first 24 h of ICU admission, which precludes the use of average values. This may have resulted in discontinuities in the smooth curve analysis. (2) In this study, HR/T was calculated based on the worst initial measurements recorded within the first 24 h of ICU admission. Consequently, the conclusions presented herein are applicable only to the worst values. Nevertheless, further research is required to investigate the mean values and dynamic fluctuations of heart rate and temperature, and additional studies are necessary to provide further evidence. (3) This study could only establish associations rather than determine causation. Meanwhile, given the intrinsic nature of our study design, several immeasurable confounding factors would inevitably arise. Accordingly, we adjusted for a range of confounding variables to ensure consistent results. For instance, the utilization of *β*-blockers may potentially impact the observed association. However, the results remained consistent after adjusting for this variable as a covariate. We used the E-value to quantify the impact of the unmeasured confounding factors and found that these factors were unlikely to significantly influence the results. (4) The diagnosis of CHF was based on the application of administrative codes. Although the first diagnostic code was used, misclassifications may have resulted in unintended associations.

In conclusion, among patients with HF admitted to the ICU, there was a significant association between HR/T ratio and 28-day ICU mortality. As the HR/T ratio increased, the risk of 28-day ICU mortality increased.

## Data Availability

The datasets presented in this study can be found in online repositories. The names of the repository/repositories and accession number(s) can be found at: https://physionet.org/content/eicu-crd/2.0/.

## Footnotes

^1^ http://www.empowerstats.com
